# First person – Pratibha Singh

**DOI:** 10.1242/dmm.046870

**Published:** 2020-08-27

## Abstract

First Person is a series of interviews with the first authors of a selection of papers published in Disease Models & Mechanisms, helping early-career researchers promote themselves alongside their papers. Pratibha Singh is first author on ‘Maltodextrin-induced intestinal injury in a neonatal mouse model’, published in DMM. Pratibha is a postdoctoral research fellow in the lab of Dr Camilia R. Martin at Harvard Medical School, Boston, MA, USA, investigating nutrient-driven immunomodulation in neonatal health and disease.


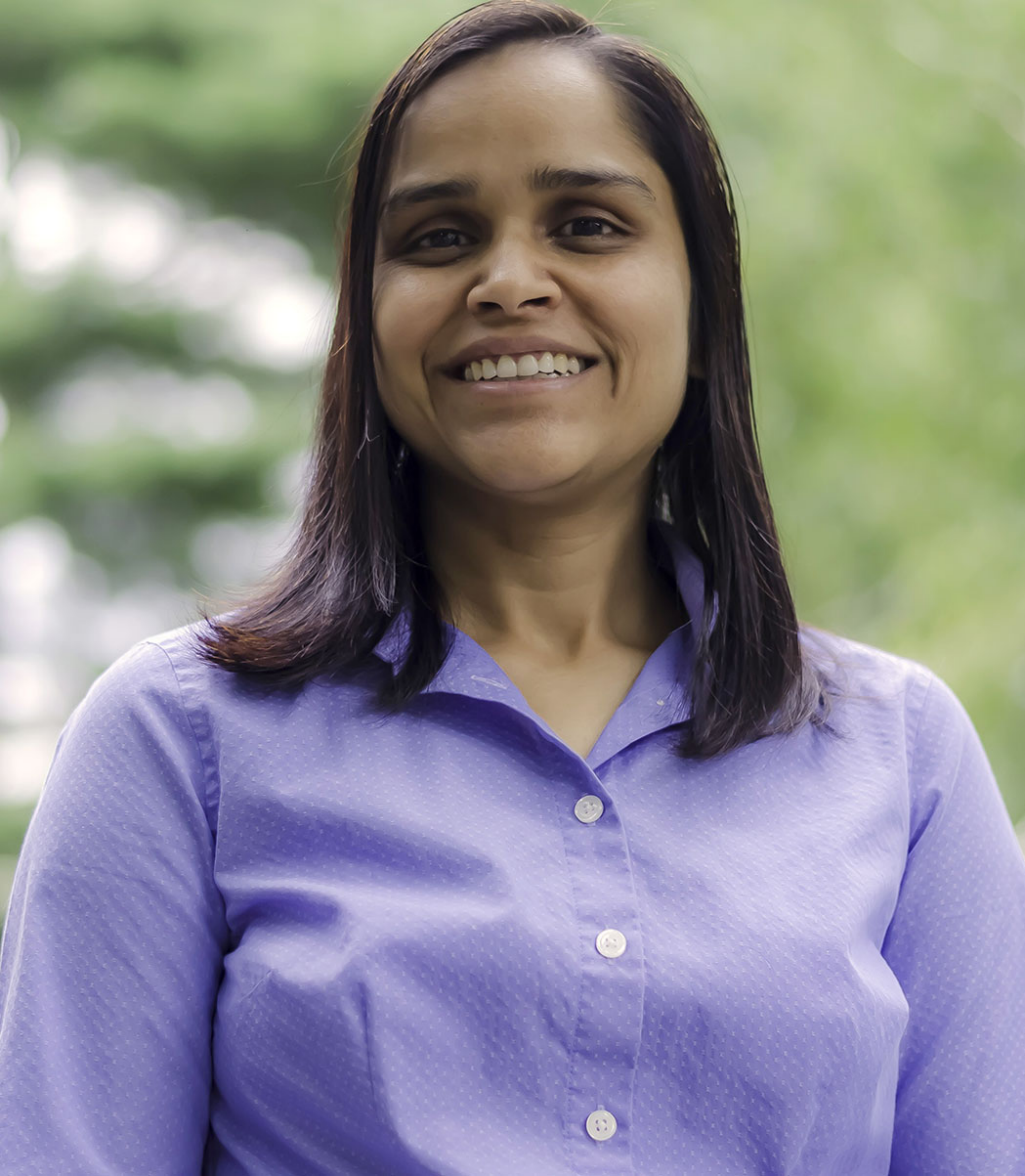


**Pratibha Singh**

**How would you explain the main findings of your paper to non-scientific family and friends?**

Necrotizing enterocolitis (NEC) is a disease of the intestines in preterm infants. NEC affects 5-12% of premature babies born below 1500 g and up to one-half will not survive. Prematurity and feeding are two major risk factors for NEC but many other factors also play an important role, making it difficult to summarize what causes NEC in a simple manner. Many animal models of NEC have been published, but the relationship of these models to what is happening in infants is variable. We hoped to develop a mouse model that was driven by feeding factors and reliably caused intestinal injury. In this study, we successfully developed an intestinal injury model in mouse neonatal pups by feeding them a formula that contains maltodextrin, a complex sugar, in combination with limited oxygen delivery after feedings. The intestinal injury in this model has several features similar to human NEC.

**What are the potential implications of these results for your field of research?**

This intestinal injury model has the potential to improve preclinical testing for new therapeutic approaches. Our intestinal injury model will also help us to explore the developmental and adaptive intestinal responses induced by early enteral priming with varying long-chain polyunsaturated fatty acids (LCPUFAs) and their subsequent effect on the risk of intestinal injury. We anticipate that this model could help to increase the clinical translation of treatments for preterm gut health.

**What are the main advantages and drawbacks of the model system you have used as it relates to the disease you are investigating?**

Our maltodextrin-induced intestinal injury model has two main advantages. The first is that, unlike previous murine models, our model is highly reproducible and has a high level of prevalence of intestinal injury. Second, our model represents a developmental model within the early postnatal period, which correlates with the timing of the highest risk in premature infants and thus provides a basis for understanding developmental vulnerability for intestinal damage. Our study has a few limitations. Even though mice share many similarities with human NEC, our study is limited by the short life span of the mouse and the fact that mice cannot be subjected to surgical intervention. Moreover, the maturation of maltodextrin digestion in human infants is not known. We acknowledge that besides maltodextrin there were subtle differences in additional components in different prespecified commercially available formulas used in our study, and the contribution of other components could not be fully ignored in this model.

**What has surprised you the most while conducting your research?**

I was surprised by the lack of a reproducible and clinically relevant animal model for NEC. NEC is a multifactorial disease and the fact that the exact etiology of the disease remains poorly understood makes it even more challenging to generate an appropriate animal model. Unlike other intestinal inflammatory diseases that largely affect the colon, NEC is a patchy disease that is most commonly found in the distal small bowel. Moreover, the experimental model of spontaneous NEC is not known. Although many labs have attempted to generate different animal NEC models (mouse, rat and piglet), these models are unable to recapitulate all the clinical features seen in human NEC, warranting further research.

**Figure:**
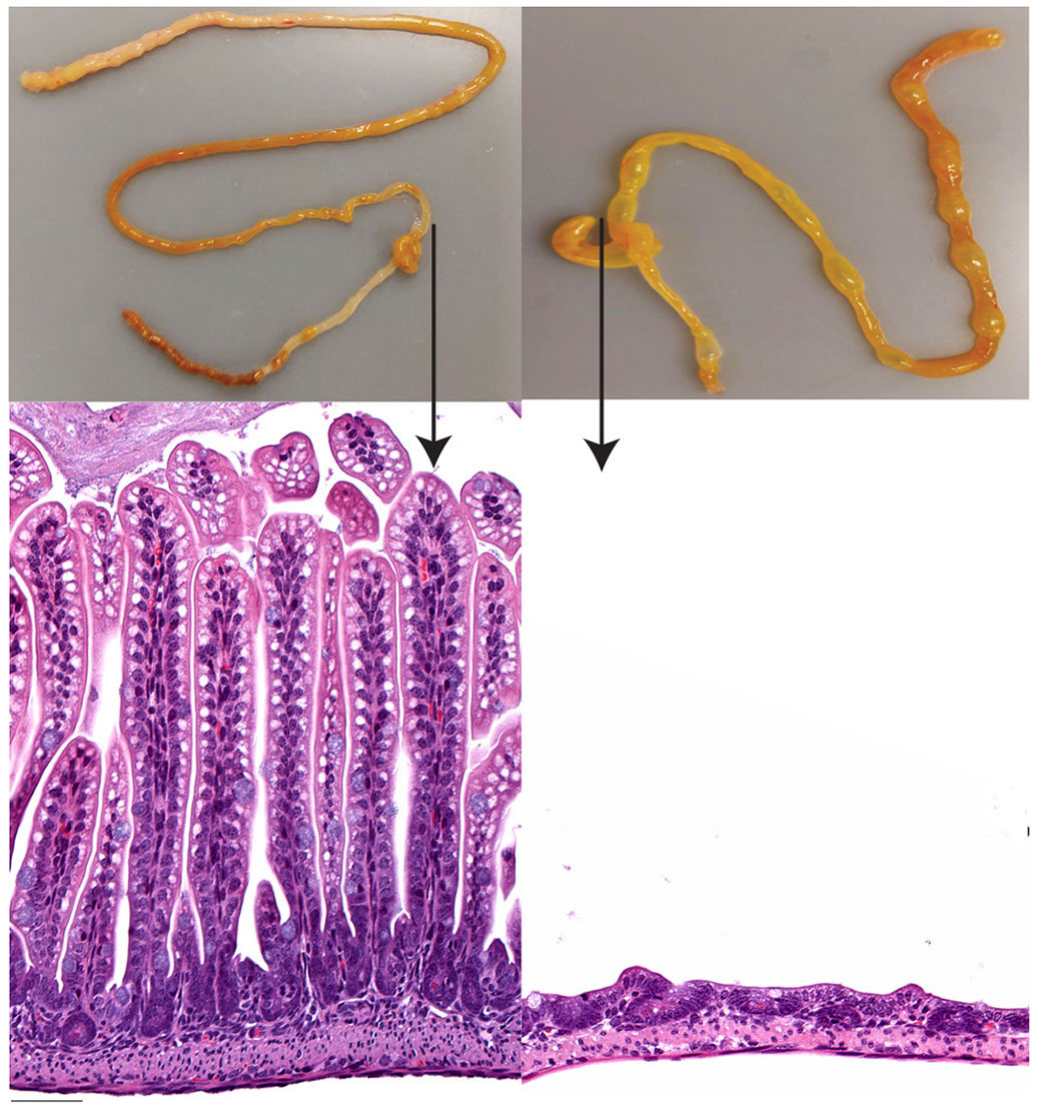
**Histology of distal ileum from mouse, showing complete loss of villi (from dilated region) as a result of intestinal injury.**

**Describe what you think is the most significant challenge impacting your research at this time and how will this be addressed over the next 10 years?**

Optimizing the delivery of fatty acids to preterm infants to improve their growth and development remains a significant challenge in clinical care. Although a few studies have shown gut developmental changes in response to altered fatty acids, there is a critical gap in complete understanding of the biological phenotype in response to changes in critical fatty acid levels and effect on neonatal health outcomes. Future experiments using this maltodextrin-induced intestinal injury model will allow us to determine the role of different fatty acid profiles in disease risk and pathogenesis. Use of this model will facilitate studies on the impact of fatty acid imbalance, specifically LCPUFAs in intestinal injury, and thus will help us understand the role of different fatty acid profiles in systemic and adaptive immune response, cellular stress and energy metabolism. Overall, these results will provide a basis to change the current standard in nutritional practices for preterm infants in the neonatal intensive care unit, by targeting specific levels of LCPUFAs. Moreover, the data generated will help identify early biomarkers reflective of changes in the host response secondary to nutritional delivery of fatty acids.

“Optimizing the delivery of fatty acids to preterm infants to improve their growth and development remains a significant challenge in clinical care.”

**What changes do you think could improve the professional lives of early-career scientists?**

The most important thing is to have excellent mentors/supervisors with clearly defined projects or important research questions that need to be answered. This focus has major influence on the professional lives of early-career scientists. Teaching how to translate their personal and professional skills within a given work place (academic or industrial setting) is pivotal. Internship programs that provide training to research fellows could also enhance many aspects of their career development. Additionally, initiating and establishing multidisciplinary collaborations through workshops, seminars and conferences could be beneficial to improve the professional lives of early-career scientists.

**What's next for you?**

I will be studying the impact of LCPUFAs in our model and unravelling the underlying mechanisms. The results from these studies will take our work to the next level in exploring the pathways by which alteration in critical fatty acids leads to neonatal disease outcomes.
